# Hydrogel-Forming Ability and Biological Characterization of Exopolysaccharide (EPS) from *Porphyridium cruentum*

**DOI:** 10.3390/gels12050352

**Published:** 2026-04-23

**Authors:** Marta M. Duarte, Artem Suprinovych, Anabela Veiga, Ana I. Lopes, Freni K. Tavaria, Rui C. Morais, Ana L. Oliveira

**Affiliations:** Universidade Católica Portuguesa, CBQF—Centro de Biotecnologia e Química Fina—Laboratório Associado, Escola Superior de Biotecnologia, Rua Diogo Botelho 1327, 4169-005 Porto, Portugal; s-msmduarte@ucp.pt (M.M.D.); s-asuprinovych@ucp.pt (A.S.); s-anveiga@ucp.pt (A.V.); anlopes@ucp.pt (A.I.L.); ftavaria@ucp.pt (F.K.T.); rcmorais@ucp.pt (R.C.M.)

**Keywords:** EPS, *Porphyridium cruentum*, hydrogel, ionic crosslinking, antioxidant, antimicrobial, immunomodulatory

## Abstract

Exopolysaccharides (EPSs) are emerging as sustainable polymers for biomedical hydrogels. Here, we report hydrogels from sulfated EPSs produced by *Porphyridium cruentum* and ionically crosslinked with Ca^2+^, Ce^3+^, or Cu^2+^ to generate tunable networks with bioactive potential. Rheological analysis showed viscoelastic behavior was primarily governed by cation nature and accessible binding site density, with diminishing gains above 2.5 wt% EPS and limited benefit beyond 10 wt% crosslinker. Ce^3+^ produced the most solid-like gel, Ca^2+^ yielded more thixotropic networks, and Cu^2+^ promoted rapid, heterogeneous crosslinking consistent with fast surface complexation. These network signatures showed distinct in vitro performances. Cation selection tuned antibacterial activity against *Staphylococcus aureus* and *Escherichia coli*, with Cu^2+^ achieving rapid bactericidal effects and Ce^3+^ enabling an 8-log reduction after 24 h. The ABTS assay showed that Ca^2+^- and Ce^3+^-crosslinked gels had antioxidant potential (≥40 µM Trolox eq.mg^−1^); however, antioxidant capacity was assay dependent. Conditioned-medium assays showed ≥75% viability at day 3 for Ca^2+^- and Ce^3+^-crosslinked gels against human dermal fibroblasts (HDFs), while only Ce^3+^-crosslinked gels were cytocompatible against human keratinocytes (HaCaTs). Cu^2+^-crosslinked gels were highly cytotoxic across all tested conditions. Macrophage cytokine readouts (TNF-α and IL-6) indicated formulation-dependent immunobiological response. This work establishes microalgal EPSs as versatile polymers and links crosslinking chemistry to rheological modulation and multifunctional biomedical performance, while direct wound-healing efficacy remains to be demonstrated in future in vivo or wound repair functional models.

## 1. Introduction

Chronic wounds remain a major clinical burden because they frequently combine excess exudate, high microbial load/biofilms, and dysregulated inflammation that delays re-epithelialization and tissue remodeling. Gel-like wound dressings have therefore become central in advanced wound care, as they can adapt to irregular wounds, maintain a moist environment, and serve as matrices for delivery of bioactive compounds. There has been a growing focus on using natural materials, such as polysaccharide-based hydrogels, as they are often biocompatible and biodegradable, meaning they can be locally absorbed, eliminating the danger of damaging the wound while removing the wound dressing [1,2].

Marine microalgae are natural sources of multifunctional biopolymers. The red microalga *Porphyridium cruentum* secretes high-molecular-weight sulfated exopolysaccharides (EPS), mainly composed of α- and β-galactose, glucose, xylose, and arabinose, with some other sugars also present [3,4,5,6]. They contain glucuronic acid and half-ester sulfates, making these molecules negatively charged with acidic properties [7] and ionic interaction potential. When prepared in aqueous media, they display high viscosity, non-Newtonian flow, and reversible thermogelation while remaining stable across broad processing conditions [3,4,7,8]. They are thought to form a weak, structured network that disperses as loosely associated microgel particles, likely driven by electrostatic interactions between positively charged protein moieties and negatively charged carboxyl/sulfate groups along the polysaccharide chains [7]. These combined physicochemical and rheological features make Porphyridium EPS promising candidates for developing hydrogel platforms for cosmetic and pharmaceutical use. Importantly for wound healing, EPSs have been linked to bioactivity relevant to the wound microenvironment, including antioxidant and immunomodulatory effects, such as antibacterial [9], antiviral [4], immunomodulatory [5,10,11], antitumor [8,12,13], and antioxidant activities [5,14,15].

Despite these attributes, translating Porphyridium EPS into clinically acceptable wound dressings requires controllable gelation under mild conditions. Ionic crosslinking is compelling because it avoids reactive covalent chemistry and can be performed in physiological conditions. Calcium-crosslinked polysaccharides, such as alginate-based wound dressings, are already well established in wound care; however, Ca^2+^-only crosslinking is often primarily structural and may not by itself address major chronic wound issues such as bacterial burden or oxidative stress [16]. In this context, cerium (Ce^3+^) offers a distinctive opportunity, as it can act simultaneously as a multivalent ionic crosslinker and as a biofunctional component. Recent studies report enhanced antibacterial and wound-healing performance compared with conventional Ca^2+^-crosslinked materials, supporting the concept that Ce^3+^ can add therapeutic function while still providing simple ionic gelation [17,18].

In this study, we analyzed the use of *P. cruentum* EPS as a marine-derived bioactive polysaccharide matrix to form hydrogels by exploiting the EPS’s native sulfated/uronic-acid chemistry to form ionically crosslinked networks under mild conditions. EPS aqueous preparations were made to interact with calcium, magnesium, iron, zinc, cerium, and copper crosslinking solutions, and the most promising EPS–cation hydrogel systems were then characterized for their post-gelling mechanics, bioactive potential, and in vitro cytocompatibility. Through this work, we sought to explore how crosslinking chemistry influences both gel mechanics, stability, and bioactive potential, including antibacterial and antioxidant activities, cytocompatibility, and immunological response. Importantly, a conventional structural polymer (alginate) and crosslinker (Ca^2+^) were directly benchmarked against potentially bioactive cations (Ce^3+^ and Cu^2+^) to elucidate formulation trade-offs. The authors hope to provide a preliminary screening of possible applications for EPS–cation hydrogel systems, with a focus on bioactive properties that are relevant to their use as a new platform for wound healing.

## 2. Results and Discussion

### 2.1. Chemical Characterization of Extracted and Purified P. cruentum EPS

The chemical and monomeric composition of *P. cruentum* EPS was obtained to better understand the mechanical and bioactive potential of these molecules. The results of this characterization are presented in Table 1.

The results show that *P. cruentum* produces heteropolysaccharides. Previous studies have reported that these EPSs are composed of three neutral monosaccharides (xylose, glucose, and galactose) and one uronic acid (glucuronic acid) [14,19]. Our results also show the presence of uronic acids at 6.7 wt%. High galactose and uronic acid content have been linked to antioxidant potential in EPSs [14,20]; furthermore, the presence of carboxylic groups further confers an overall negative charge to EPSs and is a common binding point for ionic crosslinking. Additionally, these are sulfated polysaccharides, with sulfate content increased by the enrichment of the culture media with magnesium sulfate [4]. The antibacterial and antiviral activities of *Porphyridium* EPS have likewise been correlated with the degree of sulphation [4,8].

### 2.2. Rheological Properties of EPS Solutions

**Figure 1:**
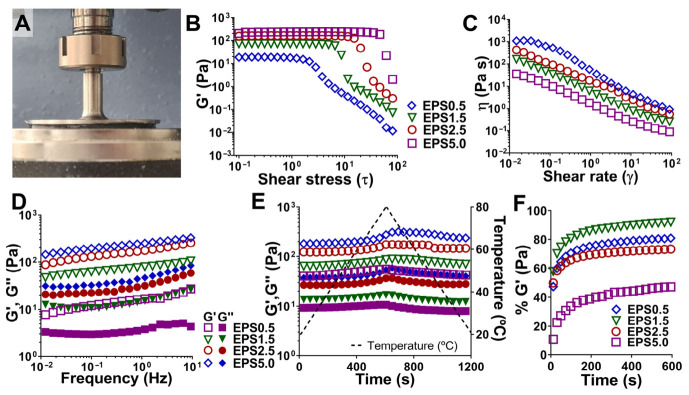
Rheological properties of *P. cruentum* exopolysaccharides (EPS) solutions. (**A**) EPS solution placed in a cone and plate geometry, ensuring optimal filling for proper rheology assessment. (**B**) Amplitude sweeps of different EPS solutions for determining the linear viscoelastic region (LVR). (**C**) Flow curves of different EPS solutions and respective application for each shear rate value region (**D**) Frequency sweeps of different EPS solutions showing changes in elastic and viscous moduli after increased frequency. (**E**) Temperature sweeps of different EPS solutions showing changes in elastic and viscous moduli after a temperature increase and decrease. (**F**) Thixotropy analysis of different EPS solutions showing the percentage recovery in the elastic modulus after a period of high shear stress. Data for each curve was selected as a representative of three independent assays.

It has been previously reported that the EPSs from *P. cruentum* have unique, and potentially useful, rheological and other mechanical properties when prepared in an aqueous medium, such as relatively high viscosity, non-Newtonian fluid-like behavior, and reversible thermal gelation upon heating [3,4,7,8]. Our own examination of the rheological properties of EPS aqueous solutions at different concentrations—0.5 (EPS0.5), 1.5 (EPS1.5), 2.5 (EPS2.5), and 5.0 (EPS5.0) wt%—are shown in Figure 1.

The results show that our EPS solutions exhibit gel-like behavior with predominant elastic behavior (G′ > G″) [21], rather than behaving as viscous liquids (Figure 1D). Additionally, no crossover point (G′ = G″) was detected throughout the tested frequency range (0.01 to 10 Hz). However, no plateau was detected for the elastic modulus, which is indicative of a weak gel-like structured network. Multiple molecular interactions, increasing with polymer concentration, could be behind this *solid-like* behavior, such as ionic attraction forces between the positively charged amino groups of the protein moiety and the negatively charged carboxyl and half-ester sulfate groups present along and between the larger carbohydrate moiety chains, as well as electrostatic repulsions between the large negatively charged sulfate groups, causing the chains along the polymer backbone to adopt extended conformations [7]. Additionally, hydrogen bonding between hydroxyl groups on adjacent chains can further stabilize the network.

As commonly observed in polysaccharide solutions, all EPS solutions exhibited shear thinning behavior, with viscosity sharply decreasing with increasing shear rate (Figure 1D). This sharp decrease is indicative of a weak gel network, where polysaccharide chain entanglement is undone by applied shear stress, which begins to align in the direction of flow [7,8,22]. While this behavior shows low mechanical stability under applied stress, shear thinning is an important property for many biomaterials, such as inks for extrusion systems, injectable gels, or medical coatings [23]. In addition, EPS solutions exhibited thixotropic behavior, with high and rapid recovery of the elastic modulus after being subjected to a high shear force, except for the highest concentration (EPS5.0) (Figure 1F). This response has been reported in previous works [7,22] and may hint towards the nature of EPS solutions, which could be made of dispersions of polymer microgel particles, which break under stress but are quickly re-established upon relaxation. The higher concentration of polymer chains, resulting in a more entangled and densely connected gel network, causes a higher resistance to breaking under flow; however, it also hinders the reorganization of chains afterwards.

Aqueous polysaccharide preparations are typically subjected to heating to facilitate dissolution. It has been previously reported that aqueous preparations of *P. cruentum* EPS thickened when heated [7], and we observed the same behavior during our own work. To better understand this behavior, EPS solutions were subjected to temperature-dependent, small-amplitude oscillatory shear measurements (Figure 1E). We can observe that at higher concentrations of EPS (in our case, beginning at 2.5 wt%), heating caused a visible increase in the elastic modulus. It was possible to reverse this increase by cooling the solution, although the highest tested concentration (EPS5.0) still retained double the initial elastic modulus value even after decreasing temperature to 20 °C. This behavior suggests that higher temperatures (beginning at approximately 60 °C) promote stronger intermolecular interactions, likely driven by enhanced hydrophobic interactions between chain molecules. Previous works have linked this thermostable behavior in polysaccharides to interactions involving methyl group and the presence of protein moieties, which through heat-induced partial unfolding end up exposing hydrophobic or charged domains that facilitate additional crosslinking points [7,24]. While these assays were conducted in a closed chamber that had been previously hydrated to prevent water loss from samples, it is important to mention that dehydration may also have contributed to this increase, since dehydration can cause changes in polymer–water interactions and to the gel’s macrostructure or lead to the loss of hydration shells around sulfate and methyl groups, favoring intermolecular hydrogen bonding and van der Waals forces [25,26,27,28].

#### Hydrogel-Forming Potential of EPS Solutions

**Figure 2:**
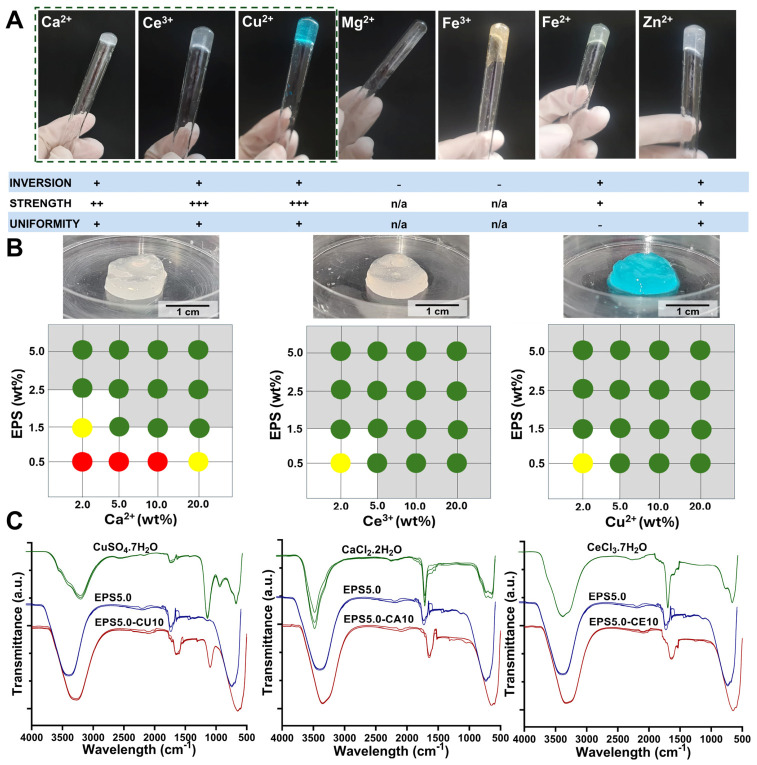
Hydrogel-forming potential of *P. cruentum* EPS. (**A**) Tube inversion test of different EPS–cation gel systems, as well as their classification according to gel strength and uniformity. (**B**) Conceptual diagrams displaying the effect of varying EPS and cation concentrations for chosen EPS–cation hydrogel systems (Ca^2+^, Cu^2+^, and Ce^3+^). Red circles represent mixtures that did not lead to a gel that did not maintain its structure after inversion, and yellow circles represent weak gels that preserved their structure after inversion but did not resist external handling with a spatula, while green circles represent gels that could be held with a spatula and were subjected to rheological characterization. (**C**) FTIR spectra (*n* = 3) obtained in the 500 cm^−1^ to 4000 cm^−1^ range for each EPS–cation hydrogel system, as well as the polymer solution and the respective cation salt used for each hydrogel preparation.

The presence of negatively charged groups, such as uronic acids and half-ester sulfates (Section 2.1), confer an overall negative charge to the EPS produced by *P. cruentum* with the potential to ionically interact with cations present in solution. To assess their hydrogel-forming potential, EPS solutions were prepared and made to interact with cation solutions at different concentrations. Both divalent cations (Fe^2+^, Cu^2+^, Ca^2+^, and Mg^2+^) and trivalent cations (Fe^3+^ and Ce^3+^) were studied. Gel formation was then assessed by visual inspection and classified according to their integrity and homogeneity. Gels that maintained their structure after tube inversion (+) and those that did not (-) were then assessed for their apparent strength after handling with a spatula (from + to +++, weakest to strongest gels, respectively) and finally if the gel appeared homogeneous (+) or not (-). The results are presented in Figure 2.

All cations tested led to at least partial gel formation, although not all gels were strong enough to resist a tube inversion assay (Figure 2A). Gel formation happened instantaneously for all cations tested, which in some cases led to difficulties in the formation of homogeneous gels. The lack of control over reaction kinetics can be a hindrance to the formation of strong and homogeneous hydrogels, as when crosslinking occurs too rapidly, polymer chains may have insufficient time to rearrange themselves into an ordered network. Previous works involving EPS hydrogels have reported that too-fast crosslinking led to phase separation and inhomogeneous gels, creating regions of dense aggregates surrounded by un-crosslinked zones, which decreased post-gelling stiffness and elasticity [29]. Heterogeneous crosslinking can result in local variations in mechanical properties, potentially compromising overall material performance, particularly at high strains [30]. Additionally, rapid surface crosslinking can slow further diffusion into the interior gel network, as well as slow the diffusion of bioactive compounds into the exterior of the gel, further affecting the gel’s biological performance [31].

This phenomenon can be controlled by promoting slow ion diffusion into the polymer mixture (e.g., through the use of a dialysis membrane). However, this may limit the range of possible applications for certain EPS–cation hydrogel systems, such as those that require in situ crosslinking. In this work, no measures to control reaction kinetics were taken other than controlling the cation concentration in the crosslinking solution, and gels were obtained after instantaneous crosslinking.

Hydrogel mechanical properties are highly influenced by the biochemical profile of the polymer in solution. The affinity of a certain polymer towards different cations is affected by the number of binding sites and their availability, which is affected due to polymer conformation [29,32]. The polyelectrolyte nature of EPS grants the polymer the ability to interact with metal ions in a range of varying affinities. Furthermore, the extraction and purification protocol used during the isolation of EPS can also have a significant effect on the affinity of the polymer to certain cations, due to contamination present in the EPS mixture such as the presence of other ions inefficiently removed from the cell culture media (e.g., Na^+^) [33]. In our study, the EPS from *P. cruentum* showed a higher affinity to calcium (Ca^2+^), cerium (Ce^3+^), and copper (Cu^2+^). Indeed, the addition of magnesium to the EPS aqueous solution did not lead to the formation of a strong gel. This lack of affinity has been previously reported in alginate gelation studies, as well as other works with EPS, reportedly due to lack of strong polymer–ion interactions [29,34]. Mg^2+^ ions have been previously described as diffusely bound counterions, in opposition with more strongly site-bound ones, such as Ca^2+^, which may help explain their nongelling properties [34]. In contrast, the strong binding affinity of EPS towards Ca^2+^, Ce^3+^, and Cu^2+^ may be primarily driven by the abundance of negatively charged and electron-donating functional groups present in EPS powders. Depending on the source and extraction procedure, EPS matrices are often a mixture of several precipitates that can include a heavy polysaccharide fraction with hydroxyls, uronic acids (that have carboxylate groups), and sulphate modifications, as well as co-extracted biomolecules such as proteins, with amine or amide, and other phenolic or aromatic groups (from humic substances) [4,6,33]. These groups provide multiple binding sites that enable ion exchange and electrostatic bridging, inner-sphere coordination/chelation for more strongly complexing ions (e.g., chelation), and, in the case of transition metals like Cu^2+^ and rare-earth metals like Ce^3+^, strong coordination bonds with oxygen and nitrogen donor atoms [33]. In the present work, the discussions of binding chemistry are proposed as plausible mechanisms based on the EPS chemical functionality and the observed rheological signatures, but direct studies of ion-binding state, spatial ion distribution, and microscopic network architecture were not examined.

Studies conducted on the adsorption behavior of sludge-derived EPS have demonstrated that carboxyl and hydroxyl groups are essential for heavy metal adsorption [35,36,37]. For copper ions specifically, it has been previously observed that their affinity to EPS is primarily driven through their interaction with amide I and amide II groups of proteins, followed by carboxyl groups in the polysaccharide fraction and finally interaction with humic acids [36]. However, because EPS composition is strongly source and purification dependent, the relative contribution of proteinaceous and humic-like fractions to metal binding may differ in purified microalgal EPS. Therefore, extrapolation from sludge EPS should be made cautiously. In this work, we have analyzed the biochemical composition of *P. cruentum* EPS and confirmed the presence of proteins, uronic acids, and sulphate in EPS biomass. For Zn^2+^, it is humic acids rather than polysaccharides that appear to be the driving force behind the affinity of some EPS matrices to this ion [36], which may explain why in our work, the EPS of *P. cruentum* did not have a high affinity towards zinc, as these groups were not present in a significant way in EPS mass. For all tested ions, polymer concentrations increased gelification, although for cerium and copper, gelification occurred even at the lowest tested polymer concentration (Figure 2B).

### 2.3. Post-Gelling Physical Properties of EPS Hydrogel Systems

**Figure 3:**
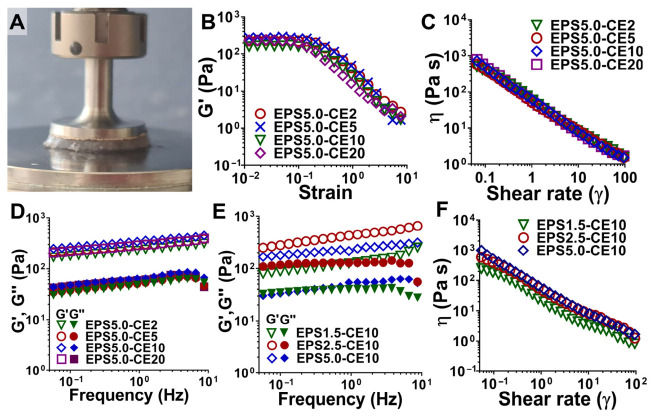
Rheological characterization of EPS–cation hydrogel systems with differing polymer and cation concentrations. (**A**) Parallel plate geometry with loaded EPS–cation hydrogel sample. (**B**) Amplitude sweeps of different EPS solutions for determining the LVR. (**C**) Flow curves and (**D**) frequency sweeps of EPS–cation hydrogels with different polymer concentrations (1.5, 2.5, and 5.0 wt%). (**E**) Frequency sweeps and (**F**) flow curves of EPS–cation hydrogels with different cation concentrations in crosslinking solution (2.0, 5.0, 10.0, and 20.0 wt%). Data for each curve was selected as a representative of three independent assays.

**Figure 4:**
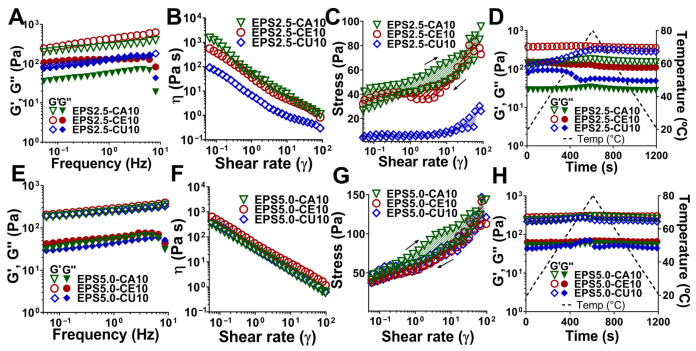
Rheological characterization of EPS–cation hydrogel systems with differing cation natures. (**A**) Frequency sweeps, (**B**) flow curves, (**C**) thixotropy analysis, and (**D**) temperature sweeps of 2.5 wt% EPS–cation hydrogels (EPS2.5-CA10, EPS2.5-CE10, and EPS2.5-CA20). (**E**) Frequency sweeps, (**F**) flow curves, (**G**) thixotropy analysis, and (**H**) temperature sweeps of 5.0 wt% EPS–cation hydrogels (EPS5.0-CA10, EPS5.0-CE10, and EPS5.0-CA20). Data for each curve was selected as a representative of three independent assays.

After the results from the inversion assay (Section Hydrogel-Forming Potential of EPS Solutions), the best performing gel formulations (calcium, cerium, and copper) were analyzed for their rheological behavior to better understand both gelling mechanics and possible future uses. The results are shown in Figure 3 and Figure 4.

#### 2.3.1. Impact of Polymer and Cation Concentration

Flow curves and frequency sweeps of EPS–cation hydrogels with different polymer concentrations (1.5 (EPS1.5), 2.5 (EPS2.5), and 5.0 (EPS5.0) wt%) and different polymer concentrations (2.0 (-CE2), 5.0 (-CE5), 10.0 (-CE10), and 20.0 (-CE20) wt%) were obtained to study the impact of polymer and cation concentration. The elastic modulus (G’) increased with the polymer concentration within the tested range (Figure 3D), likely due to the increasing polymer chain density and subsequent entanglement. This increase has been previously observed with EPS-based gels [29] and for alginate and related ionically crosslinked polysaccharide hydrogels [38,39]. Our results show that there is no increase in the elastic modulus when the polymer concentration is increased beyond 2.5 wt%, since the values of the elastic modulus were higher for EPS2.5-CE10 (Figure 3E). As explained above, the affinity of a polymer towards a cation is affected by the number of available binding sites. It is possible that a denser network may increase the number of binding sites but reduce its availability as well. Also, while the sulphate groups of EPS increase their affinity to positively charged groups, they also may hinder the creation of a tightly bound and dense network, as sulphate groups repel each other electrostatically, an effect already reported by previous groups working with ι-carrageenan [29,40]. It is also possible that crosslinking kinetics may play a part, as less homogeneous crosslinking may occur with a higher polymer concentration, and surface crosslinking may hinder the diffusion of cations into the hydrogel network [29]. However, the elastic modulus for EPS5.0-CE10 was less frequency dependent than that observed in EPS2.5-CE10 and EPS1.5-CE10, which may indicate a more tightly bonded hydrogel network [41]. Weaker or more loosely crosslinked gels often show a sharp rise in G′ as the frequency increases, in contrast to a flatter G′ line, because they are initially viscous dominated at low frequencies but become stiffer at higher oscillation rates, as molecular chains, crosslinks, or entangled structures do not have time to properly rearrange or dissipate energy. It is important to mention that frequency-dependent behavior may be desirable for some gels, as opposed to those with more mechanical integrity. While the latter is usually desired for load-bearing applications, the former is more beneficial for injectable gels or those used for extrusion-based techniques, especially at high frequency rates [23].

In contrast, increasing the concentration of the cation in the crosslinking solution appeared to have minimal effect after a certain threshold (5.0–10.0 wt%), as shown by the lack of a significant increase in the elastic modulus between EPS5.0-CE5, EPS5.0-CE10, and EPS5.0-CE20 (Figure 3D). This appears to indicate that the limiting factor in the crosslinking reaction is the polymer concentration, as after a certain wt% is achieved, the number of available binding sites becomes more limited, and it becomes necessary to add more polymer to capture more cations. The availability of binding sites is also affected by ion diffusion into the main gel mass, which again can be affected by uneven crosslinking, which is more likely to happen at high polymer or crosslinker concentrations [29,42]. Instead, it was the nature of the cation used that had a more significant effect on the rheological behavior of EPS–cation hydrogel systems.

#### 2.3.2. Impact of Polymer and Cation Nature

The nature of the cation used (Ca^2+^, Ce^3+^, and Cu^2+^) for the crosslinking solution had a visible effect on the mechanical properties of both 2.5 (EPS2.5) and 5.0 (EPS5.0) wt% EPS–cation hydrogel systems (Figure 4A,B).

Hydrogel stiffness, as measured by the elastic modulus, was highest for cerium-based gels, followed by calcium. Although *P. cruentum* EPS has a high affinity for copper, as demonstrated above (Section Hydrogel-Forming Potential of EPS Solutions), the lack of control over reaction kinetics led to the formation of a heterogeneous gel that was stiff in the outer boarder but more liquid-like inside. This was easily observed with the EPS2.5-CE10 gel, as the G’ and G’’ had very similar values, with G’ being only slightly above G’’, although no crossover point was detected within the tested frequency range. This high affinity for copper, which leads to excessively fast crosslinking kinetics and uneven gel networks, has previously been reported by other groups for gels involving both EPS and alginate [29,32]. In our work, this effect was more pronounced in 2.5 wt% (Figure 4A) than 5.0 wt% EPS gels (Figure 4E), where all the gels (EPS5.0-CA10, EPS5.0-CE10, and EPS5.0-CU10) showed similar values for the elastic modulus and similar frequency-independent behavior for all cations used, likely due to the higher binding site availability leading to more crosslinking points and the denser polymer chain network.

**Figure 5:**
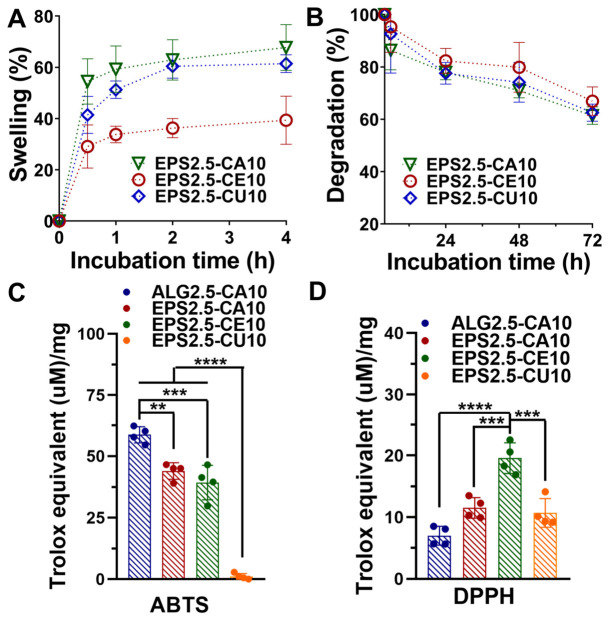
Swelling and degradation behaviors of EPS–cation hydrogel systems. (**A**) Swelling (*w*/*w* %) and (**B**) degradation (*w*/*w* %) of EPS2.5-CA10, EPS2.5-CE10, and EPS2.5-CU10 gels. Data is represented as the mean ± SD of five replicates. (**C**) Scavenging effect of ALG2.5-CA10, EPS2.5-CA10, EPS2.5-CE10, and EPS2.5-CU10 gels as measured with ABTS and (**D**) DPPH assays. Data is represented as the mean ± SD of four replicates. One-way ANOVA was used for overall comparisons, and Tukey’s multiple comparisons test was applied between different experimental groups (** *p* < 0.01, *** *p* < 0.001, and **** *p* < 0.0001).

To evaluate the gel’s thixotropic behavior, we performed a hysteresis loop measurement (Figure 4A,B). Calcium-crosslinked gels exhibited a higher thixotropy area than cerium and copper gels, which may signify a less tightly bound gel network. This area represents the extent of structural breakdown and subsequent recovery of the hydrogel under shear. As previously mentioned, under high shear, the hydrogel suffers from polymer chain disentanglement, which can lead to the collapse of the polymer–cation network when these forces are stronger than the affinity of binding forces [23]. A high thixotropy area indicates that either greater structural disruption occurred or that the network is slower in rebuilding its microstructure under the tested conditions [23]. This effect was observable in both 2.5 wt% (Figure 4C) and 5 wt% (Figure 4G) gels, where calcium-crosslinked gels had a significantly higher area than cerium- and copper-crosslinked gels.

Temperature sweeps (Figure 4D,H) also revealed that EPS–cation hydrogel systems behave differently depending on the crosslinking cation. For EPS–cation hydrogels, an increase in temperature led to a stiffer material, as previously reported for EPS solutions (Section 2.2), although the cation-crosslinked hydrogels showed less temperature-dependent behavior than EPS solutions and recovered quickly as temperature decreased. However, for both cerium- and copper-crosslinked gels, an increase in temperature led to a decrease in the elastic modulus (and increase in the viscous modulus), which was especially more pronounced in copper-linked gels. These differences may be attributed to the types of bonds at play for each cation. Calcium has previously been reported in other work to interact electrostatically with carboxylate and sulfate groups in EPS, forming a relatively labile “egg-box” network. These bonds, while not rigid or strong, are not temperature sensitive, such as those observed in alginate–calcium gels, which show thermal stability up to approximately 100 °C [32]. In contrast, copper ions form stronger, more directional coordination complexes with a broader range of EPS functional groups, including amines from proteins, as well as carboxylates and hydroxyl groups from the polysaccharide network [35,36,37]. These bonds are stronger than those observed with calcium and result in a more rigid initial network, which is also more thermally sensitive, as temperature increases lead to increased ligand exchange dynamics and possible conformational changes in the EPS polymer chain network. The covalent nature of these bonds means they are also less quick to recover after this disruption. It is worth mentioning that there are uses for both thermally sensible and thermal-stable gel types, as the former is essential for extrusion-based printing.

### 2.4. In Vitro Properties of EPS Hydrogel Systems

#### 2.4.1. Swelling and Degradation Rates

After obtaining thresholds for both the EPS (2.5 wt%) and cation (10.0 wt%) concentration, after which an increase leads to diminishing returns according to rheological analysis (Section 2.3.1), the in vitro behaviors of EPS2.5-CA10, EPS2.5-CE10, and EPS2.5-CU10 were analyzed. The swelling and degradation rates are presented in Figure 5.

All gels showed a rapid initial hydration ramp followed by a plateau (at ~2 h); however, the swelling degree varied in response to the cation used, with EPS2.5-CE10 showing the lowest swelling percentage (~33%) compared to the remaining two (~60–62%). Several factors can contribute to the capacity of hydrogels to retain water (or other aqueous solutions), such as the existence of hydrophilic groups within the polymeric network or the degree and rigidity of the crosslinking network. Ce^3+^, having a higher charge than Ca^2+^ and Cu^2+^, can promote a tighter ionic network and higher effective crosslink density, which reduces chain mobility and limits water uptake [42]. The degradation profile appears to further support this interpretation, as although all gel formulations showed similar degradation throughout the 72 h assay period, EPS2.5-CE10 exhibited a lower degradation percentage, although the difference was smaller than the one observed in swelling percentage. It is important to note that since gelation occurred essentially instantaneously, it is not possible to exclude a degree of influence from crosslinking heterogeneity in these results.

#### 2.4.2. Antibacterial and Antioxidant Potential

Antioxidant data for EPS2.5-CA10, EPS2.5-CE10, EPS2.5-CU10, and ALG2.5-CA10 was obtained through ABTS and DPPH assays (Figure 5C,D). Both Ca^2+^-crosslinked gels, ALG2.5-CA10 and EPS2.5-CA10, showed the highest antioxidant activity (>40 Trolox Equivalents (µM)/mg), while EPS2.5-CU10 showed no antioxidant effect. Importantly, the EPS used in these formulations has high sulfate, galactose, and uronic acid content, which has been correlated with antioxidant potential [14,20]. Cerium-based biomaterials have also been previously reported to have antioxidant properties, driven by redox cycling between the 3+ and 4+ states [43]. These results differ from those obtained through the DPPH assay, where both Ca^2+^-crosslinked gels exhibited very low activity, and EPS2.5-CE10 showed the highest (although still below 20 Trolox Equivalents (µM)/mg). This difference may be explained by the chemistry behind ABTS and DPPH assays. ABTS and DPPH are assays where antioxidant potential is mainly driven by hydrogen atom transfer (HAT) or single-electron transfer (SET). However, they differ in radical structure, solvent environment, kinetics, and sensitivity to steric effects, with the ABTS assay being widely used for the determination of the antioxidant activity of hydrophilic and lipophilic compounds [44]. It is therefore possible that the ABTS assay may be better suited to access the antioxidant potential of the EPS-rich hydrogel phase, while the DPPH assay shows only cation-dependent redox chemistry. However, this hypothesis was not studied further, and it is therefore impossible to conclude that differences are caused by the method used. Similar assay-dependent behavior has been reported in polymeric hydrogels, where “blank” matrices show measurable ABTS activity but weaker signals in other assays, whereas antioxidant-loaded systems typically show a much stronger (e.g., ~900 to 5000 Trolox Equivalents (µM)/mg) and more concordant multi-assay responses [45,46,47].

**Figure 6:**
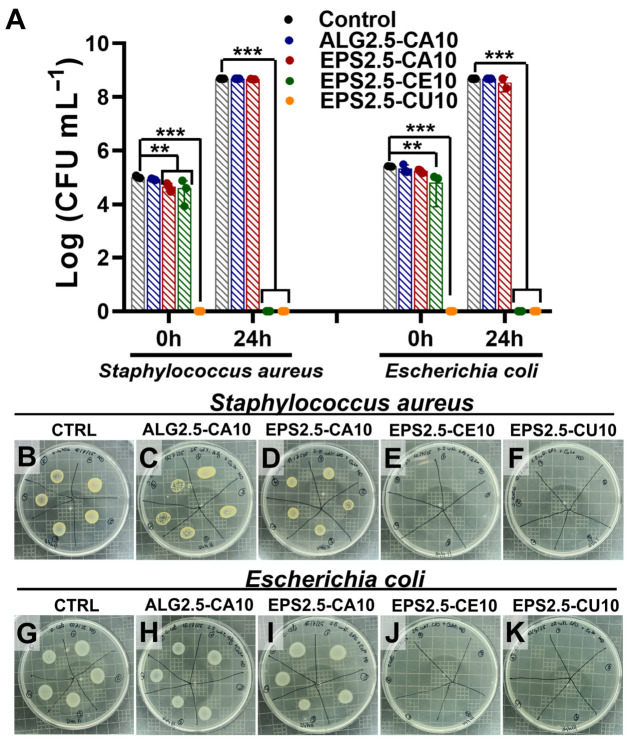
Bioactive properties of EPS–cation and model hydrogel systems. (**A**) Antibacterial properties of ALG2.5-CA10, EPS2.5-CA10, EPS2.5-CE10, and EPS2.5-CU10 against *S. aureus* and *E. coli.* (**B**–**F**) Mueller–Hinton agar plates of control (untreated), ALG2.5-CA10, EPS2.5-CA10, EPS2.5-CE10, and EPS2.5-CU10 dilutions with *S. aureus* and (**G**–**K**) *E. coli* after 24 h of incubation. Data is represented as the mean ± SD of four replicates. One-way ANOVA was used for overall comparisons, and Dunnett’s multiple comparisons test was applied between the control and different experimental groups (** *p* < 0.01, and *** *p* < 0.001).

Regarding the antibacterial activity of the assayed gels (Figure 6A), both EPS2.5-CE10 and EPS2.5-CU10 had a bactericidal effect against *Staphylococcus aureus* (ATCC 6538) and *Escherichia coli* (ATCC 8731) growth, achieving complete inhibition of growth after 24 h for both microorganisms, while EPS2.5-CA10 showed no antibacterial effect. These results appear to indicate that for these gels, the antibacterial activity is predominantly driven by the bioactive metal ions (in this case, both Cu^2+^ and Ce^3+^) rather than the effect of the polymer used or the mechanism of gelation. Nevertheless, the present results do not imply that the EPS matrix has no antibacterial properties on its own. Negatively charged polysaccharides have been previously reported to interact with Gram-positive bacteria, possibly by interfering with and damaging the cell membrane or by disrupting biofilm-mediating signaling pathways [48,49]. However, reports on the antibacterial properties of *P. cruentum* EPS have varied. Raposo et al. found no antibacterial effect for 1 wt% EPS solutions, in contradiction to other works [4,50].

Additionally, Cu^2+^-crosslinked gels achieved complete inhibition of growth of both bacterial populations after 0 h, showing a strong bactericidal effect. This early-onset bactericidal effect is consistent with the literature on copper’s antibacterial mode of action (described as “contact killing”), in which available Cu ions at the material–bacteria interface disrupt and permeabilize cell envelopes, accumulate intracellularly, and promote oxidative stress via reactive oxygen species, leading to the loss of viability [51,52]. Also, cerium-crosslinked alginate films have been previously reported to have antibacterial activity against both *E. coli* and *S. aureus* [53]. Additionally, cerium (III) nitrate has long been used as a treatment for burn wounds [53,54] and therefore has a precedent for being used in chronic wound management.

#### 2.4.3. Cytocompatibility with HDF and HaCaT Cell Lines

**Figure 7:**
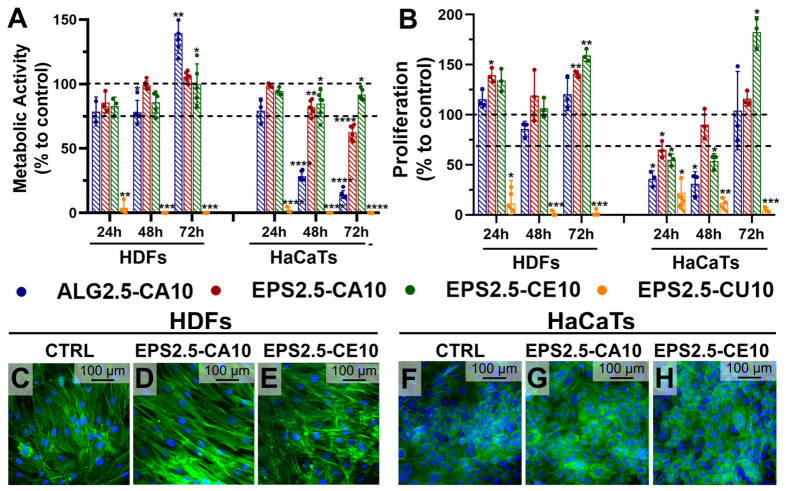
Cytocompatibility of ALG2.5-CA10, EPS2.5-CA10, EPS2.5-CE10, and EPS2.5-CU10. (**A**) Metabolic activity as measured by resazurin assay and (**B**) cell proliferation as measured by BrDU assay in HDF and HaCaT cell lines. (**C**–**E**) DAPI and phalloidin staining of HDFs and HaCaTs (**F**–**H**) treated with untreated media (control) or media conditioned with EPS2.5-CA10 or EPS2.5-CE10 at 72 h. For metabolic activity and proliferation assays, data is represented as the mean ± SD of six replicates. One-way ANOVA was used for overall comparisons, and Dunnett’s multiple comparisons test was applied between the control and different experimental groups (* *p* < 0.05, ** *p* < 0.01, *** *p* < 0.001, and **** *p* < 0.0001).

Cytocompatibility of the EPS2.5-CA10, EPS2.5-CE10, and EPS2.5-CU10 hydrogels was evaluated using conditioned media (lixiviates) in relevant skin-associated cell models, namely human dermal fibroblasts (HDFs) and human keratinocytes (HaCaTs), and the results are shown in Figure 7. Fibroblasts and keratinocytes play complementary roles in wound healing and tissue repair, with fibroblasts contributing to extracellular matrix deposition and remodeling and keratinocytes being essential for re-epithelialization and barrier restoration [55]. These cell types therefore provide a suitable in vitro platform for preliminary screening of the in vitro properties of EPS-based hydrogel formulations intended for regenerative and wound-healing related applications.

Cell viability was first assessed indirectly by measuring metabolic activity via the resazurin assay (Figure 7A) and through a proliferation assay via BrdU incorporation (Figure 7B) using gel-conditioned media (lixiviates), which primarily evaluates the biological impact of soluble, leachable species released into the medium rather than direct cell–material contact. For HDFs, all assayed formulations (with the exception of EPS2.5-CU10) caused a slight decrease in metabolic activity when compared to untreated cells (control); however, this activity was still higher than 75% and reached 100% after 72 h, which may indicate that the decrease in metabolic activity was due to an adaptation period to changes to the composition of the culture media. This decrease was not observed, however, in the proliferation percentage of the cells, which remained similar to untreated cells. In contrast, EPS2.5-CU10 caused a marked reduction in HDF metabolic activity at all time points, indicating a strong cytotoxic effect under the tested conditions. While a similar trend was observed for HaCaT cells, EPS2.5-CE10 showed the most favorable profile, maintaining metabolic activity and proliferation near control values over time, while EPS2.5-CA10 and ALG2.5-CA10 formulations were shown to have a cytotoxic effect, although less pronounced for the EPS formulation. A possible explanation for these results is that HaCaTs are highly calcium responsive, and their proliferation and differentiation balance is strongly regulated by extracellular Ca^2+^ [56]. The calcium released from Ca2+-crosslinked gels to the conditioned media may therefore have a higher effect on HaCaT growth dynamics than HDFs. It is important to mention that these results should not be over-interpreted as evidence of favorable cell adhesion on the gel surface or as definite proof that the gels are not cytotoxic but rather as a preliminary screening of ionic/leachable effects under the selected extraction conditions.

Again, EPS2.5-CU10 induced near-complete loss of metabolic activity, indicating poor cytocompatibility toward keratinocytes in this assay format. These results are in accordance with the literature, where a concentration- and time-dependent cytotoxic effect of soluble copper compounds on HaCaT cells has been previously reported [57]. Overall, EPS2.5-CU10 gels were consistently cytotoxic to both HDFs and HaCaTs, making Cu^2+^-crosslinked gels unsuitable as wound-dressing candidates in their current form.

To further evaluate the effect of EPS–cation hydrogel formulations on both HDF and HaCaT cell lines, cell morphology was assessed through fluorescence microscopy (Figure 7C–H). The evaluation of cell morphology appears to confirm the results from the quantitative cytocompatibility assays, with HDFs retaining their characteristic elongated morphology, with uniformly distributed and intact nuclei in all samples, supporting the conclusions of cell viability (Figure 7C–E). While HaCaTs retained their polygonal, cobblestone-like appearance, the number of nuclei present in the image with cells treated with EPS2.5-CA10 appears to be lower when compared to untreated and EPS2.5-CE10 samples (Figure 7F–H).

#### 2.4.4. Immunomodulatory Effect on THP-1 Cell Lines

**Figure 8:**
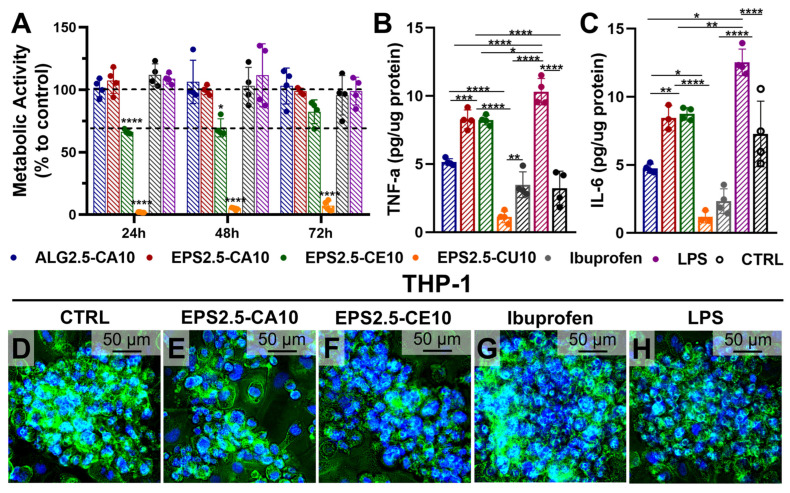
Immunomodulatory effect of ALG2.5-CA10, EPS2.5-CA10, EPS2.5-CE10, and EPS2.5-CU10. (**A**) Metabolic activity as measured by resazurin assay in THP-1-differentiated macrophage-like cells. (**B**) TNF-α and (**C**) IL-6 production after stimuli in THP-1-differentiated macrophage-like cells. (**D**–**H**) DAPI and phalloidin staining of THP-1-differentiated macrophage-like cells treated with untreated media (control) or media conditioned with EPS2.5-CA10, EPS2.5-CE10, ibuprofen, or LPS at 72 h. Data is represented as the mean ± SD of six replicates for metabolic activity and of four replicates for cytokine levels. One-way ANOVA was used for overall comparisons, and Dunnet’s multiple comparisons test was applied between the control and different experimental groups, while Tukey’s multiple comparisons test was applied between different experimental groups (* *p* < 0.05, ** *p* < 0.01, *** *p* < 0.001, and **** *p* < 0.0001).

As for the immunomodulatory effect on THP-1 cells (Figure 8), EPS2.5-CA10 and EPS2.5-CE10 showed distinct immunobiological effects. EPS2.5-CA10 maintained macrophage metabolic activity near control levels, while EPS2.5-CE10 produced a moderate reduction, again possibly reflecting an adaptation period due to changes to the media culture composition. EPS2.5-CU10-treated cells again showed strong cytotoxicity, which may explain the low cytokine levels observed for this formulation.

Regarding cytokine secretion, both EPS2.5-CA10 and EPS2.5-CE10 increased TNF-α and IL-6 relative to untreated and ALG2.5-CA10 controls, which may be evidence of an early pro-inflammatory profile, although the present dataset is insufficient to determine whether this response is transient and potentially pro-regenerative or persistently detrimental. Currently, none of the tested EPS-based formulations can be described as clearly anti-inflammatory under the conditions evaluated. However, in wound healing, a moderate early pro-inflammatory macrophage response is not inherently negative, as TNF-α and IL-6 are characteristic of early inflammatory-phase macrophage activation [58,59]. However, whether this response is compatible with a pro-regenerative immunological response depends on its magnitude and, critically, its resolution over time, parameters not captured in the current dataset. Also, because this assessment was performed in a single macrophage-like cell model (THP-1) and with a limited cytokine panel evaluation, the present data should be interpreted as a preliminary study on immunomodulatory potential rather than concrete evidence of beneficial immunoregulation for wound healing. Overall, the results suggest that cation identity can also modulate the immune response to EPS-based hydrogels, with possible implications for their potential applications.

## 3. Conclusions

The results of this work demonstrate that *P. cruentum* EPS can be formulated into ionically crosslinked hydrogel-like materials with tunable rheological and biological properties. Cation nature rather than concentration was the main determinant of rheological performance once a minimum crosslinker concentration was reached, suggesting that gel formation became constrained by polymer-site accessibility and diffusion-limited crosslinking rather than ion availability. Among the tested ions, Ce^3+^ produced the more tightly connected gel structure, and Ca^2+^ gels were more thixotropic and more shear labile, indicating a softer and more recoverable network, while Cu^2+^ gels showed evidence of fast, non-homogeneous crosslinking, which compromised network uniformity.

The gels also showed clear cation-dependent bioactive properties. Cu^2+^-crosslinked gels exhibited the strongest antibacterial activity; however, under the tested conditions, this was accompanied by pronounced cytotoxicity in HDFs, HaCaTs, and THP-1 cells, indicating that Cu^2+^ gels, as currently formulated, are not realistic wound-dressing candidates. Ca^2+^-crosslinked gels had the highest swelling capacity; however, they showed no antibacterial properties and induced reduced HaCaT responses, supporting their classification as comparatively benign but functionally limited matrices under the tested conditions. In contrast, Ce^3+^-crosslinked gels showed the best overall balance, combining favorable swelling and degradation behavior, strong antibacterial activity, and the most consistent cytocompatibility and proliferative support for both fibroblasts and keratinocytes, thereby providing the most favorable overall in vitro balance of mechanical integrity, antibacterial performance, and cytocompatibility in vitro among the tested formulations.

Importantly, the current assays and focus of this study was to provide a preliminary screening of wound-relevant properties, and functional wound-healing models (e.g., direct-contact assays, scratch/migration assays, and in vivo wound models) were not included in this work. Therefore, future work should seek to include these assays and to investigate long-term stability and ion release behavior under more realistic wound bed conditions as to substantiate wound-healing efficacy.

## 4. Materials and Methods

### 4.1. Materials

*P. cruentum* strain was kindly provided by Allmicroalgae-Natural Products, S.A, Pataias, Portugal. Thricloroacetic Acid (>99%), ethanol (≥99.5%), cellulose membrane (MWCO = 12,000, size 33 mm × 21 mm), resazurin sodium salt, nitric acid (60%), trifluoroacetic acid (TFA, 99%), sodium nitrate (≥99.0%), sodium bicarbonate (≥99.5%, molecular biology), sodium dihydrogen phosphate dihydrate (≥99.0%), RPMI-1640 medium, and paraformaldehyde (95%, powder) were obtained from Sigma-Aldrich (St. Louis, MO, USA). Chloroform (≥99.9%) and sulfuric acid (97.0%) were purchased from Honeywell (Charlotte, NC, USA). Premium CO_2_ Liquid Premier (99.995%) was obtained from Gasin Air Products (Perafita, Portugal). The Pierce™ Bradford Protein Assay Kit and 2-Mercaptoethanol were obtained from Thermo Fisher Scientific (Waltham, MA, USA). The Cell Proliferation ELISA BrdU kit was obtained from Roche Diagnostics (Mannheim, Germany). Human dermal fibroblasts (HDFs) were obtained from Innoprot (Bizkaia, Spain). The human keratinocyte cell line (HaCat) was acquired from Cell Line Services (Oppenheim, Denmark). Dulbecco’s Modified Eagle’s Medium (DMEM) was purchased from Gibco™ (Thermo Fisher Scientific). Fetal bovine serum (FBS) was obtained from BioWest (Nazaré, Portugal). Penicillin−streptomycin was purchased from Lonza (Basel, Switzerland). Flash Phalloidin™ Green 488, 4′,6-Diamidino-2-Phenylindole, Dilactate (DAPI), and the ELISA MAX^TM^ Deluxe Set (IL-6 and TNF-α) were purchased from BioLegend (San Diego, CA, USA). Mueller–Hinton broth and agar were purchased from Biokar Diagnostics (Allonne, France). THP-1 (TIB-202™) cells, *Staphylococcus aureus*, and *Escherichia coli* were obtained from American Type Culture Collection (ATCC, Manassas, VA, USA).

### 4.2. Culture Conditions of Porphyridium Cruentum

Culture conditions were as described by Raposo et al. [4], with some modifications (Supplementary Materials Section S1.1). Axenic cultures of P. cruentum were grown in a walk-in chamber, under continuous light (30 μEm^−2^ s^−1^) and constant temperature (25 °C). The cultures were kept under constant agitation by gently bubbling compressed air into the culture flasks through sterilized 0.2 μm filters (Ø 55 mm). The composition of culture media is described in Table 1. The medium was enriched with MgSO_4_ to lead to increased EPS production and sulfate content [4]. For the determination of culture collection point, samples were taken from each replicate every two days for pH measurement and determination of biomass dry weight. The cultures were collected in the early stationary phase.

### 4.3. Extraction and Purification of EPS

#### 4.3.1. Separation from Biomass

The cultures were centrifuged at 10,000 rpm for 30 min at 4 °C. The supernatant, containing the cell-free culture media and EPS, was collected and further processed, while the pellet containing the microalgal biomass was discarded.

#### 4.3.2. Deproteinization via Trichloroacetic Acid (TCA)

TCA was added to the supernatant with a final concentration of 5% (*v*/*v*) to denature and precipitate the proteins present in solution. The solution was then homogenized at 37 °C for 40 min and centrifuged (8000 rpm, 4 °C, 15 min) to remove any remaining protein. The supernatant was collected and further purified, while the pellet was discarded.

#### 4.3.3. Ethanol Precipitation

The supernatant was placed in a water bath at 80 °C for 1 h and then filtered with a simple filter paper to remove suspended particles. Cold EtOH (90%) was added to the supernatant in a proportion of vol 1:1, under constant stirring. The mixture was then left at −20°C overnight (~16 h). After precipitation, the EPS were extracted from the mixtures by centrifugation (8000 rpm, 15 min, 4 °C). The supernatant was discarded, and the pellet (with EPS) was collected and further purified.

#### 4.3.4. Dialysis

Dialysis was performed by resuspending ethanol-precipitated EPS in deionized water and dialyzing with a cellulose membrane (MWCO = 12,000) against deionized water, several times.

#### 4.3.5. Supercritical-CO_2_ Drying (scCO_2_)

**Figure 9:**
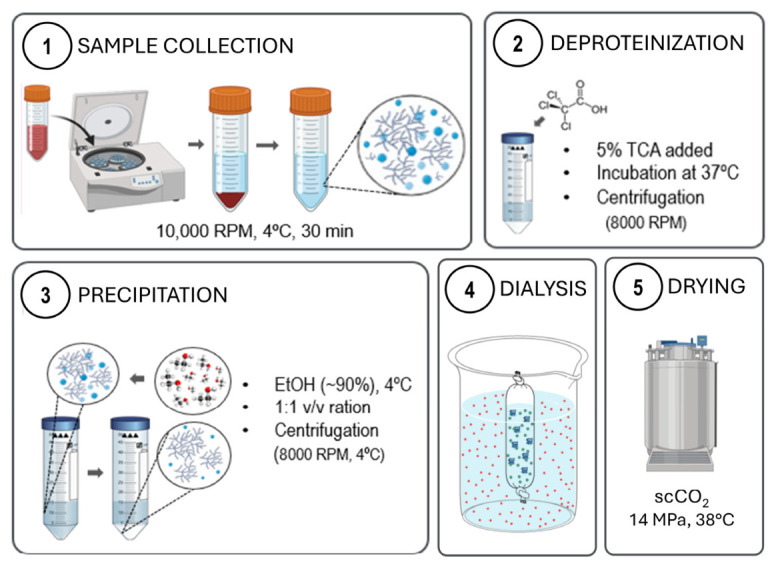
Schematic representation of EPS processing and purification. Created with BioRender.com.

The dialyzed EPS samples were first placed in a series of ethanol solutions (70%, 80%, 90%, and 100% *v*/*v*) for solvent replacement, sealed into sterilization pouches (Tyvek, DuPont, DE, USA), and loaded inside the pressure vessel of a Parr Instruments series 4540 high-pressure reactor (Parr Instrument Company, Moline, IL, USA). Liquid CO_2_ was pumped into the system via a high-pressure pump at 50 g/L until a pressure of 14 MPa at 38 °C and 600 rpm. The equipment was operated in semi-continuous mode using a constant CO_2_ flow of 20 g/min. After 45 min, the vessel was depressurized slowly (~3.5 bar/min) using a manually operated valve. Dried EPS was then milled using a mortar and pestle and stored in a desiccator for later use. A schematic representation of sample collection, extraction, and purification is found in Figure 9.

### 4.4. EPS Composition

#### 4.4.1. Carbohydrates (Neutral Oses) and Acid Sugars

Carbohydrates (as neutral oses) were determined colorimetrically through the phenol–sulfuric acid method [60], with some modifications (Supplementary Materials Section S1.2). Total uronic acids were determined colorimetrically through the carbazole method [20], with some modifications (Supplementary Materials Section S1.3).

#### 4.4.2. Proteins and Lipids

Protein content was determined colorimetrically using a Pierce™ Bradford Protein Assay Kit using the kit recommendations. Total lipid content was determined through the chloroform method [61], with some modifications (Supplementary Materials Section S1.4).

#### 4.4.3. Sulphate

Sulfate content was obtained turbidimetrically, as barium sulfate, according to the method described by Dodgson and Price [62] and adapted by Torres et al. [63].

### 4.5. Hydrogel-Forming Capacity of EPS

#### 4.5.1. Preparation of EPS Solutions

Dried EPS powder was dissolved in ultrapure water at 0.5 (EPS0.5) 1.5 (EPS1.5), 2.5 (EPS2.5), and 5.0 (EPS5.0) wt% at approximately 60 °C under constant mixing and then homogenized using a dual-syringe Luer-lock system. Solutions were then allowed to cool to room temperature until further use.

#### 4.5.2. Inversion Assay

The gel-forming capacity of EPS solution in standard conditions was assayed by preparation of cation-mediated gels using divalent cations (FeSO_4_·7H_2_O, CuSO_4_·5H_2_O, CaCl_2_·2H_2_O, and MgCl_2_·6H_2_O,) and trivalent cations (FeCl_3_·6H_2_O and CeCl_3_.7H_2_O). The gelation studies were performed according to the procedure described by Shimada et al. [64], with minor modifications: A total of 0.5 mL of EPS solution (5.0 wt%) was added to the metal salt (25 mg of cation) and agitated until dissolution, and gel formation was assessed visually by means of a tube inversion test. Gels were categorized according to their strength and homogeneity—(+) for gels that maintained their gel structure in a tube inversion test and (−) for gels that did not sustain their structure in the inversion test— and then assessed for their apparent strength after handling with a spatula from + to +++, from the weakest to strongest gels, respectively. And finally, we determined if the gel appeared homogeneous (+) or not (−).

#### 4.5.3. Model and EPS–Cation Hydrogel Systems

EPS–cation hydrogels were obtained via ionic crosslinking using the divalent and trivalent cations chosen after the inversion assay (CuSO_4_·5H_2_O, CaCl_2_·2H_2_O, and CeCl_3_.7H_2_O). EPS solutions at 1.5, 2.5, and 5.0 wt% were prepared as described in Section 4.5.1 and then crosslinked with either 2.0, 5.0, or 10.0 wt% of either Ca^2+^, Ce^3+^, Cu^2+^ in salt form. A list of all formulations with labels and the concentrations of EPS and of each cation is shown in Table 2.

The post-gelling rheological, mechanical, and biological behaviors of EPS hydrogels were compared to those of a well-characterized biopolymer in the literature, alginate. Alginate hydrogels were dissolved in ultrapure water at 2.5 and 5 wt% at approximately 60 °C and then allowed to cool to room temperature before being crosslinked with a 10 wt% calcium chloride (CaCl_2_·2H_2_O) solution.

### 4.6. Rheological Characterization of EPS Solutions and Hydrogels

#### 4.6.1. EPS Solutions

Rheological measurements were conducted with an air-bearing Bohlin Instruments Gemini Advanced Rheometer (Bohlin Instruments, Cirencester, UK), connected to a Peltier for controlling system temperature operated with Bohlin software (GEMINI 150, v6.50.5.8). The rheological behavior of EPS wt% solutions was assayed using a cone-and-plate geometry (40 mm diameter, 4° cone angle, 100 µm gap), All assays were conducted at room temperature. Amplitude sweeps were measured logarithmically by an increased shear stress amplitude from 0.1 to 100 Pa at a fixed frequency of 1 Hz. Amplitude sweeps were used to determine the linear viscoelastic (LVE) region, which was determined as 0.5 Pa for all following assays. Frequency sweep tests were conducted at constant shear stress amplitude from 0.01 to 10 Hz. Flow curves were obtained via a logarithmic shear rate ramp test between 0.01 and 100 (1/s). Temperature sweeps were conducted at constant shear stress with a frequency of 1 Hz. The samples were subjected to a temperature ramp from 20 °C to 80 °C followed by cooling to 20 °C. To prevent sample dehydration, the assays were conducted within a closed and previously humified chamber. The thixotropic behavior of the samples was evaluated in a three-stage oscillatory shear test. During the first stage, the sample was measured within the previously determined LVE region followed by a high oscillatory shear of 100 Pa for 5 s. The recovery of the structure was then measured over 10 min within the LVE region. In total, 100 data points were collected for all the assays.

#### 4.6.2. EPS–Cation Hydrogels

The rheological behavior of EPS–cation hydrogels was assayed as above (Section 4.6.1) with the following differences: The assay was conducted using a parallel plate geometry (20 mm diameter, 1000 µm gap), All assays were conducted at room temperature. Amplitude sweeps were measured logarithmically using a strain ramp from 0.01 to 10 at a fixed frequency of 1 Hz. Amplitude sweeps were used to determine the linear viscoelastic (LVE) region, which was determined as 0.1 for all following assays. Frequency sweep tests were conducted at constant strain from 0.01 to 10 Hz. Temperature sweeps were conducted at constant strain with a frequency of 1 Hz. The samples were subjected to a temperature ramp from 20 °C to 80 °C followed by cooling to 20 °C. To prevent sample dehydration, the assays were conducted within a closed and previously humified chamber. Flow curves and thixotropic behavior were obtained via a logarithmic shear rate ramp test that happened within two stages: first, a ramp from 0.01 and 100 (1/s) was used and then from 100 to 0.01 again.

### 4.7. In Vitro Characterization of EPS–Cation Hydrogel Systems

#### 4.7.1. Swelling

The swelling ratio of EPS–cation hydrogels was measured gravimetrically. Hydrogel samples (*n* = 4) were dried via an air oven (60 °C) for 1 h and weighed (*w*_0_) and then placed in cell strainers (1 µm porosity) and immersed in PBS (pH = 7.4) at 37 °C. At specific time points (*w_t_*, t = 0.5, 1, 2, 4, and 6 h), the wet weight of the samples was measured after absorbing the excess water with the help of a filter paper. At each time point (*w_t_*), the swelling ratio was calculated according to the following equation (Equation (1)):

(1) $$swelling\;ratio\left(\%\right)=\frac{w_{t}-w_{0}}{w_{0}}\times 100$$

where *w_t_* is the wet weight of the sample’s tested for the different time points, and *w*_0_ is the initial dry weight of the hydrogel.

#### 4.7.2. Degradation

The in vitro degradation of EPS–cation hydrogels was analyzed gravimetrically under simulated physiological conditions. Hydrogel samples (*n* = 4, θ 6 mm, 2 mm height) were immersed in PBS (pH = 7.4) and incubated at 37 °C under constant agitation. Cell strainers with 1 µm porosity were used to support each hydrogel sample. The initial wet weight (*w*_0_) of each sample was measured after 4 h of incubation; then, the weight of each hydrogel sample was measured at specific time periods (*w_t_*, t = 1, 2, 4, 8, 16, 24, and 72 h). Before weighing, samples were gently blotted with filter paper to remove the excess liquid. The degradation ratio at the time point was calculated using the following Equation (2):

(2) $$Degradation\left(\%\right)=\frac{w_{0}-w_{t}}{w_{0}}\times 100$$

where *w*_0_ is the initial wet weight of the hydrogel, and *w_t_* is the wet weight tested at each point.

#### 4.7.3. Antimicrobial Activity

The antibacterial activity of EPS–cation hydrogels was determined by adapting the viable cell method as described by Lopes et al. [47,65]. Before the test, an overnight liquid culture of *Staphylococcus aureus* (ATCC 6538) and *Escherichia coli* (ATCC 8731) was prepared in Mueller–Hinton broth, and the optical density was adjusted to approximately 0.13 at λ = 625 nm, which corresponds to about 10^8^ CFU mL^−1^. Then, the liquid cultures were diluted in Mueller–Hinton broth to an inoculum concentration of 10^5^ CFU mL^−1^. EPS–cation hydrogels were prepared under sterile conditions, cut into 1 cm discs, and distributed into a 48-well plate, where 100 µL of *S. aureus* or *E. coli* inoculum was placed over each disc. The hydrogel samples were left in contact with the inoculum for 0 and 24 h at 37 °C. After incubation, 400 µL of peptone water was added to each tube, and the solution was homogenized until the hydrogel was completely dissolved; then, 4 ten-fold dilutions of each sample were undertaken in sterile peptone water. An aliquot (~20 µL) of each dilution was pipetted onto Mueller–Hinton agar plates and incubated at 37 °C for 24 h, and afterwards, the colonies were counted. The results were expressed as the mean log CFU mL^−1^ of three replicates of each hydrogel.

#### 4.7.4. Antioxidant Activity

The antioxidant activity of EPS–cation hydrogels was evaluated using the ABTS and DPPH assays according to Gonçalves et al., with a few modifications [66]. The ABTS radical (ABTS+) was generated by a reaction of 2,2′-Azino-Bis (3-ethylbenzothiazoline-6-sulfonic acid) (7 mM) with potassium persulfate 2.45 mM, which was stirred overnight. The ABTS stock solution was filtered (0.45 μm) and diluted in pure ethanol to an absorbance of 0.70 (±0.02) at 734 nm for the control. 2,2-difenil-1-picrilhidrazil (DPPH) was dissolved in methanol to a concentration of 600 μM and then diluted in ethanol to an absorbance of 0.600 (±0.100) at 515 nm.

Each hydrogel was cut into approximately 20 mg pieces, which were placed into 2 mL tubes. Then, 2 mL of ABTS or DPPH was pipetted into each tube. The tubes with ABTS and DPPH were left to reach in the dark at RT for 5 min and 30 min, respectively. After the incubation time, the solutions’ absorbance was measured in a spectrophotometer Shimadzu UV mini-1240 (Shimadzu, Kyoto, Japan) at 734 nm (ABTS) and 515 nm (DPPH). Trolox solutions in a range of 0–2500 µM were used to obtain the standard curve. The results were expressed as Trolox Equivalents per mg of gel (Trolox Equivalents (M)/mg gel). All analyses were performed in quadruplicate.

### 4.8. In Vitro Evaluation of Cytocompatibility of EPS–Cation Hydrogel Systems

#### 4.8.1. Culture Conditions

HDFs were used between passages 20 to 30, while HaCaTs were used between passages 30 to 40. HDFs and HaCaTs were cultured in DMEM (high glucose), supplemented with 10% fetal bovine serum (FBS), and 1% of penicillin−streptomycin (P/S) solution. THP-1 cells were cultured in RPMI 1640 media supplemented with 2-mercaptoethanol (to a final concentration of 0.05 mM), 10% FBS, and 1% P/S. Cultures were maintained at 37 °C in a humidified 5% CO2 atmosphere. HDFs and HaCaTs were allowed to grow until 70–90% confluency, while THP-1 grew to 1.5 × 10^6^ cells/mL.

#### 4.8.2. Cell Viability Assay

The cytotoxicity of EPS–cation hydrogels was evaluated indirectly via a lixiviate assay using conditioned culture media, with HDF and HaCaT cells, following ISO 10993-5:2009 guidelines [67]. HDFs and HaCaTs were first seeded in 96-well plates to assess cell viability at a density of 0.5 × 10^4^ cells per well. Metabolic activity was assayed colorimetrically using a resazurin assay, while cell proliferation was monitored through BrdU incorporation via an in situ detection kit, following kit instructions.

Cells were incubated for 24 h at a 37 °C in a humidified 5% CO_2_ atmosphere. Culture medium (10 mL) was conditioned for 24 h with 2 g of sterile hydrogel samples (EPS2.5-CA10, EPS2.5-CE10, EPS2.5-CU10, and ALG2.5-CA10), at 37 °C under constant agitation. After initial incubation, the cell medium was replaced with the conditioned media and left to incubate for 24, 48, and 72 h. The cells in the BrdU plates were also incubated with 10 µL of BrdU labeling working solution. For the resazurin assay, resazurin sodium salt was first dissolved in PBS at a stock concentration of 1 mg/mL and then dissolved in DMEM at 0.1 mg/mL. At each time point, the medium with EPS was replaced with resazurin working solution and left to incubate for 2 h in the dark, after which the fluorescence at an excitation wavelength of 560 nm and an emission wavelength of 590 nm was read using a Biotek Synergy H1 microplate reader (Agilent Technologies, Santa Clara, CA, USA). For the BrdU assay, the absorbance of the samples was read after 8 min of incubation with the kit’s substrate solution at 370 nm, with a reference wavelength 492 nm. The study included negative and positive controls, and blanks of DMEM were used between passages 20 to 30, while HaCaTs were used between passages 30 to 40. HDFs and HaCaTs were cultured in DMEM (high glucose) supplemented with 10% fetal bovine serum (FBS) and 1% penicillin−streptomycin (P/S) solution. THP-1 cells were cultured in RPMI 1640 media supplemented with 2-mercaptoethanol (to a final concentration of 0.05 mM), 10% FBS, and 1% P/S. Cultures were maintained at 37 °C in a humidified 5% CO2 atmosphere. HDFs and HaCaTs were allowed to grow until 70–90% confluency, while THP-1 grew to 1.5 × 10^6^ cells/mL.

#### 4.8.3. Immunomodulatory Assay

THP-1 cells were seeded in 96-well plates at near-confluent density (1.0 × 10^6^ cells/mL) and differentiated into macrophage-like cells by co-incubation with phorbol-12-myristate-13-acetate (PMA, 50 ng/mL) for 48 h at 37 °C in a humidified 5% CO_2_ atmosphere. After differentiation, wells were washed with PBS, and cells were stimulated with lipopolysaccharide (LPS) at 1 µg/mL in complete culture medium for 24 h. Following LPS stimulation, cells were washed with PBS and incubated with either conditioned media as described above (Section 4.8.2), 1 µg/mL LPS, 50 µg/mL ibuprofen, or complete culture medium. After 24 h, cell culture supernatants were collected and centrifuged (200× *g*, 10 min, 4 °C) to remove cellular debris, and clarified supernatants were stored for subsequent cytokine quantification at −80 °C. Cells were then washed with cold PBS and lysed using 200 µL cold distilled water and stored at −20 °C for downstream protein quantification.

Human TNF-α and IL-6 levels were quantified using ELISA MAX™ Deluxe Set assays (BioLegend, San Diego, CA, USA), following kit instructions, while total protein was quantified using a Micro BCA Protein Assay (Thermo Fisher Scientific Inc., Waltham, MA, USA). Results were expressed in pg of TNF-α and IL-6 per µg of protein.

#### 4.8.4. Cell Morphology

Cell morphology was analyzed via fluorescence microscopy. For both analysis, cell coverslips were inserted into a 24-well plate, which were then seeded with HDF and HaCaT cells (seeding density of 0.05 × 10^6^ cells per well) and exposed to cultured media as described above (Section 4.8.2). At each time point, the cover slips were washed with PBS, fixed for 30 min in 4 wt% paraformaldehyde, and then washed and subsequently stored in PBS at 4 °C. Afterwards, samples were permeabilized with 0.5% Triton X-100 for 10 min, washed with PBS, and then incubated for 30 min with 5 wt.% FBS in PBS. Samples were incubated with the Phalloidin™ Green 488 at a dilution of 1:100 in PBS for 45 min at RT to stain F-actin. After staining, samples were washed two times with PBS, and nuclei were counterstained with DAPI at a concentration of 300 nM for 5 min. Fluorescence imaging was performed using an inverted microscope Axio Vert A1 FL (ZEISS, Oberkochen, Germany), equipped with an LED light source and the X-Cite Xylis (D) illumination system. The microscope was configured for GFP and DAPI detection using the GFP channel (Excitation BP 470/40, beam splitter FT 495, and emission BP 525/50) and DAPI channel (Excitation G 365, beam splitter FT 395, and emission BP 445/50), respectively. Images were captured using a ZEISS Axiocam 305 color digital camera and processed with ZEN Microscopy Software v. 3.13 (ZEISS, Oberkochen, Germany).

### 4.9. Statistical Analysis

Results were expressed as the mean ± standard deviation (SD) or as representative of at least three independent experiments. For assays where *n* = 3, a Kruskal–Wallis test followed by Dunnett’s multiple comparisons test was used for comparison of the distinct experimental groups. For *n* > 3, normal distribution was tested via a Shapiro–Wilk test, followed by analysis of variance (ANOVA test) with Tukey’s HSD’s multiple comparisons. A difference between experimental groups was considered statistically significant whenever the *p*-value was <0.05 (* *p* < 0.05, ** *p* < 0.01, *** *p* < 0.001, and **** *p* < 0.0001). Statistical analyses were performed using GraphPad Prism v. 9.3.1 (GraphPad Software, La Jolla, CA, USA).

## Figures and Tables

**Table 1:** Content of carbohydrates, proteins, lipids, and sulphate in *P. cruentum* exopolysaccharides (EPS) mass (*n* ≥ 3).

Component	EPS (*w*/*w* %)
Neutral sugars	38.6 ± 3.9
Uronic acids	6.7 ± 0.8
Proteins	11.3 ± 0.9
Lipids	4.8 ± 0.2
Sulphate	6.0 ± 1.2

**Table 2:** Labels used for different EPS–cation hydrogel systems based on their composition.

Concentration (wt%)			Label
EPS	Cation		
5.0	Ca^2+^	10.0	EPS5.0-CA10
	Ce^3+^	20.0	EPS5.0-CE20
		10.0	EPS5.0-CE10
		5.0	EPS5.0-CE5
		2.0	EPS5.0-CE2
	Cu^2+^	10.0	EPS5.0-CU10
2.5	Ca^2+^	10.0	EPS2.5-CA10
	Ce^3+^	10.0	EPS2.5-CE10
	Cu^2+^	10.0	EPS2.5-CU10
1.5	Ce^3+^	10.0	EPS1.5-CE10

## Data Availability

Data is contained within the article and available upon request.

## Supplementary Materials

The following supporting information can be downloaded at: https://www.mdpi.com/article/10.3390/gels12050352/s1, Table S1. Composition of adapted Artificial Sea Water (ASW) media for *P. cruentum* culture.

## Abbreviations

The following abbreviations are used in this manuscript:

EPS	Exopolysaccharide
ABTS	2,2′-azino-bis(3-ethylbenzothiazoline-6-sulfonic acid
DPPH	2,2-diphenyl-1-picrylhydrazyl
TNF-α	Tumor necrosis factor-alpha
IL-6	Interleukin-6
LVR	Linear viscoelastic region
BrdU	5-bromo-2′-desoxiuridina
HDF	Human dermal fibroblast
HaCaT	Human immortalized keratinocyte
DAPI	4′,6-diamidino-2-phenylindole
scCO_2_	Supercritical carbon dioxide
FBS	Fetal bovine serum
