# Supplementary material for: Hydrogel-Forming Ability and Biological Characterization of Exopolysaccharide (EPS) from Porphyridium cruentum
Source: Gels. 2026 Apr 23;12(5):352. doi: 10.3390/gels12050352 (PMC13205223; doi:10.3390/gels12050352)
Supplement: Supplementary file 1 [file gels-12-00352-s001.zip › gels-4202677-supplementary.pdf]

S1.1 Composition of Artificial Sea Water (ASW) media

Composition of Artificial Sea Water (ASW) media for *P. cruentum* culture supplemented with $MgSO_4$.

Table S1. Composition of adapted Artificial Sea Water (ASW) media for *P. cruentum* culture.

For 1 L of deionized water				
**Major salts**		**Micronutrients**		**Vitamins**
		1 mL of the following solution		1 mL of the following solution
NaCl	23.6	H_3BO_3	0.568	Thiamine (1 mg/mL)
$MgSO_4.7H_2O$	12.8	$ZnCl_2$	0.624	100 µL of the following solution
$MgCl_2.6H_2O$	4.1	$CuCl_2.2H_2O$	0.268	D(+)- H-Biotin (20 µg/mL)
$CaCl_2.2H_2O$	1.46	$Na_2MoO_4. 2H_2O$	0.252	B12 (10 µg /mL)
KCl	0.075	$CoCl_2. 6H_2O$	0.42	
KNO_3	0.303	$FeSO_4.7H_2O$	2.49	
$Na_2EDTA.2H_2O$	0.05	$MnCl_2. 4H_2O$	0.36	
Tris HCl	1.0	Na.K-tartrate	2.92	
		Deionized water	1L	

S1.2 Phenol-sulfuric acid method

In-detail description of the phenol-sulfuric acid method to determine total carbohydrate content.

For the total carbohydrates content, the Phenol-Sulfuric Acid colorimetric method is performed following the method described by (Dubois et al., 1951) with some modifications. EPS samples were dissolved in ultrapure water (0.5 mg/mL) and then diluted until a final concentration of 0.1 mg/mL. 200 µL of each sample was placed in a microtube and mixed with 600 µL of concentrated sulfuric acid (95.0 - 97.0 %, Honeywell, Wabash, IN, USA). Then, 120 µL of phenol (Sigma-Aldrich, St. Louis, MO, USA) working solution (PWS 5%) was added to the mixture and heated for 5 minutes at 90 ºC in a static water bath. The solution was then allowed to cool to room temperature for 5 min. Two 200 µL sample replicates, as well as standard solutions, were transferred to a 96-well microplate. After mixing slightly, the plate was read using a Multidetection plate reader (Synergy H1, Vermont, USA) at 490nm. Glucose standards were prepared by dissolving 10mg of D-(+)-Glucose (Sigma-Aldrich, St. Louis, MO, USA) in 10ml of pure water, STD0 (1mg/mL), and then diluted into a series of concentrations from 0.5 to 0.015 mg/mL (total of 8 STDs).

S1.3 Carbazole method

In-detail description of the carbazole method to determine total uronic acid content.

A sulfamic acid (Sigma-Aldrich, St. Louis, MO, USA) 4M solution was prepared using ultra pure water, with pH 1.6 (using KOH solution). A concentrated sulfuric acid (95.0 - 97.0 %, Honeywell, Wabash, IN, USA) solution was prepared containing 75 mM of disodium tetraborate (Sigma-Aldrich, St. Louis, MO, USA). Finally, a 0.125% carbazole (Sigma-Aldrich, St. Louis, MO, USA) solution in absolute ethanol (Honeywell, Wabash, IN, USA) was prepared. To different wells of 96 well microtiter plate, 50 µl of EPS solutions and standard (galacturonic acid, concentration range of 200–1.562 µg/well) were placed in dilutions. Then, 20 µl of sulfamic acid reagent (4M, pH 1.6) was added and mixed thoroughly. To this mixture, 200µl of concentrated sulfuric acid (98%) with disodium tetraborate was added, then agitated for 1 minute. The plate was then kept in a boiling water (100ºC) bath for 15 min, followed by cooling at ambient temperature for 20 min. Then, 50 µl of the carbazole solution was added, and kept in boiling water (100ºC) bath for an additional 10 min, followed by cooling at ambient temperature for 15 min. The plated was then read with a Multidetection plate reader (Synergy H1, Vermont, USA) at a wavelength of 550 nm.

S1.4 Chloroform method

In-detail description of the chloroform method to determine lipid content.

A solution of 50 mL of 50 mM Potassium Phosphate dibasic was prepared by dissolving 0.435 g of Potassium Phosphate dibasic (Sigma-Aldrich, St. Louis, MO, USA) in 50 mL of ultrapure water and adjust to pH 7.4. 50 mg of dried EPS powder was placed in a 50 mL tube form were dissolved, where 2.5 mL of chloroform (Honeywell, Wabash, IN, USA), 5 mL of methanol (Honeywell, Wabash, IN, USA), and 2 mL of 50 mM potassium phosphate buffer were added. The mixture was left to incubate at room temperature and under agitation for 2h. Afterwards, 2.5 mL of chloroform and 2.5 mL of the potassium phosphate buffer were added into the above system again, mixed uniformly, and then stood still until layers formed within the solution. The liquid layered in the lower layer (chloroform layer) was then transferred to a previously weighted (m_1) dry glass tube. Then, this glass tube with the liquid was placed into a water bath at 60 °C, until the liquid volatilized completely, and then weighted again with dried powder (m_2). The total lipid content (LC) was calculated as follows:

$$\mathrm{LC}\left(\frac{\mu\mathrm{g}}{\mathrm{mg}}\right)\mathrm{DW} = \frac{m_2 - m_1}{50}$$
